# A Vesicle‐to‐Worm Transition Provides a New High‐Temperature Oil Thickening Mechanism

**DOI:** 10.1002/anie.201609365

**Published:** 2017-01-10

**Authors:** Matthew J. Derry, Oleksandr O. Mykhaylyk, Steven P. Armes

**Affiliations:** ^1^ Dainton Building, Department of ChemistryThe University of SheffieldSheffield, South Yorkshire, S3 7HFUK

**Keywords:** block copolymers, morphology transition, nanoparticles, polymerization-induced self-assembly, RAFT polymerization

## Abstract

Diblock copolymer vesicles are prepared via RAFT dispersion polymerization directly in mineral oil. Such vesicles undergo a vesicle‐to‐worm transition on heating to 150 °C, as judged by TEM and SAXS. Variable‐temperature ^1^H NMR spectroscopy indicates that this transition is the result of surface plasticization of the membrane‐forming block by hot solvent, effectively increasing the volume fraction of the stabilizer block and so reducing the packing parameter for the copolymer chains. The rheological behavior of a 10 % w/w copolymer dispersion in mineral oil is strongly temperature‐dependent: the storage modulus increases by five orders of magnitude on heating above the critical gelation temperature of 135 °C, as the non‐interacting vesicles are converted into weakly interacting worms. SAXS studies indicate that, on average, three worms are formed per vesicle. Such vesicle‐to‐worm transitions offer an interesting new mechanism for the high‐temperature thickening of oils.

Small‐molecule amphiphiles such as surfactants are well‐known to self‐assemble in aqueous solution to form spherical micelles,^1^ lamellae or vesicles (liposomes).^2^ In 1976, Israelachvili and co‐workers introduced the concept of a geometric packing parameter for surfactants,^3^ thus allowing their morphology to be predicted based on the relative dimensions of the hydrophilic and hydrophobic components. In 1995, Eisenberg and co‐workers^4^ reported the first examples of block copolymer vesicles using highly asymmetric polystyrene‐poly(acrylic acid) diblock copolymers. Self‐assembly occurred on addition of water, a non‐solvent for polystyrene, to a dilute copolymer solution in DMF. Bates and co‐workers reported the formation of well‐defined block copolymer worms in aqueous solution using poly(ethylene oxide)‐polybutadiene diblock copolymers.^5^ Antonietti and Förster subsequently extended the packing parameter concept to include block copolymer spheres, worms and vesicles.^6^ More recently, polymerization‐induced self‐assembly (PISA) has provided a versatile and convenient route for the synthesis of block copolymer spheres, worms or vesicles at relatively high copolymer concentrations in either aqueous,^7^ alcoholic^8^ or non‐polar^9^ media. Typically, reversible addition‐fragmentation chain transfer (RAFT) dispersion polymerization is utilized to chain‐extend a soluble macromolecular chain transfer agent (macro‐CTA) with a miscible monomer to form an insoluble polymer block, which drives in situ self‐assembly. Transmission electron microscopy (TEM) studies have shed new light on the nature of the worm‐to‐vesicle transition that can occur under these conditions, which proceeds via a “jellyfish” intermediate.^10^ PISA formulations have enabled the *rational* synthesis of low‐polydispersity vesicles using a binary mixture of two macro‐CTAs^11^ and the precise mechanism of vesicle growth during such syntheses has been recently elucidated.10a, 12

PISA enables the convenient preparation of thermoresponsive diblock copolymer nano‐objects. For example, a worm‐to‐sphere transition occurs on cooling poly(glycerol monomethacrylate)‐poly(2‐hydroxypropyl methacrylate) (PGMA‐PHPMA) diblock copolymer nanoparticles, because the core‐forming PHPMA block becomes more hydrated at lower temperatures.^13^ In contrast, worm‐to‐sphere transitions are observed on heating certain alcoholic and non‐polar PISA formulations.^13^, ^14^ In both cases, the ingress of solvent into the cores increases the effective volume fraction of the stabilizer block and hence triggers the morphological transition. Such worm‐to‐sphere transitions lead to degelation, because the multiple inter‐particle contacts formed by the anisotropic worms cannot be maintained by the isotropic spherical nanoparticles.

In the present study, we report a thermally triggered vesicle‐to‐worm transition for a PISA formulation in a non‐polar solvent (mineral oil). Small‐angle X‐ray scattering (SAXS), rheology and ^1^H NMR studies confirm that this order–order transition leads to much higher viscosity at elevated temperature, which suggests a unique oil‐thickening mechanism.

A low‐polydispersity poly(stearyl methacrylate) (PSMA; mean degree of polymerization=13) macro‐CTA was chain‐extended via RAFT dispersion polymerization of benzyl methacrylate (BzMA) monomer in mineral oil to generate well‐defined PSMA_13_‐PBzMA_96_ diblock copolymer vesicles at 10 % w/w solids (Scheme 1). A BzMA conversion of 96 % was obtained for this PISA formulation within 5 h at 90 °C, as judged by ^1^H NMR spectroscopy. THF GPC analysis confirmed that the resulting PSMA_13_‐PBzMA_96_ diblock copolymer chains exhibited a relatively narrow molecular weight distribution (*M*_w_/*M*_n_=1.17). Moreover, the clear shift in the molecular weight distribution curve relative to that of the PSMA_13_ macro‐CTA indicated a high blocking efficiency (see Figure S1 in the Supporting Information). TEM images indicate a well‐defined vesicular morphology, see Figure 1 a. SAXS studies conducted on the 5.0 % w/w dispersion of PSMA_13_‐PBzMA_96_ nanoparticles at 20 °C (red data, Figure 2) indicated a gradient of approximately −2 at low *q*, as expected for vesicles, with characteristic local minima corresponding to the outer vesicle dimensions (*q*≈0.05 nm^−1^) and the vesicle membrane thickness, *T*_m_ (*q*≈0.5 nm^−1^).

**Figure 1:**
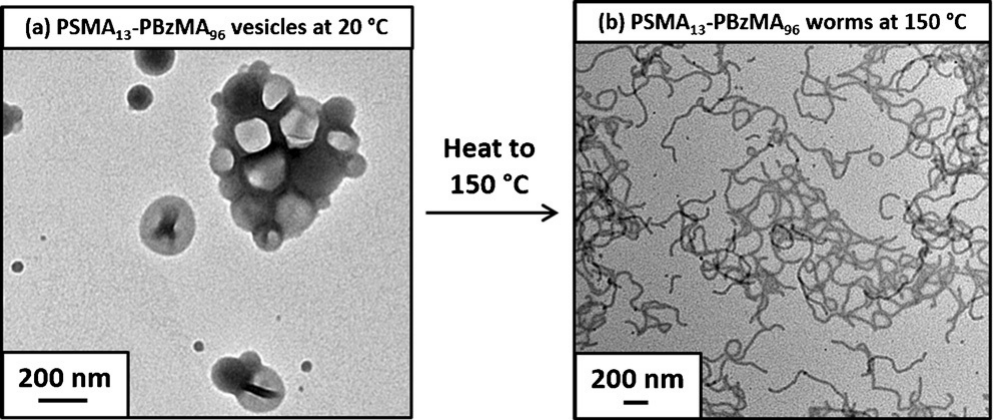
Transmission electron micrographs obtained for a) 0.1 % w/w PSMA_13_‐PBzMA_96_ vesicles at 20 °C and b) the highly anisotropic worms formed by the same vesicle dispersion on heating up to 150 °C.

**Figure 2:**
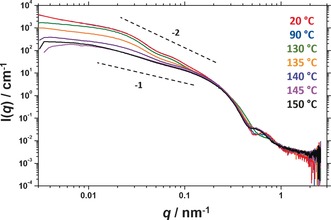
Variable temperature small‐angle X‐ray scattering (SAXS) patterns recorded for a 5.0 % w/w dispersion of PSMA_13_‐PBzMA_96_ nano‐objects in mineral oil. Gradients of −2 and −1 are shown as a guide to the eye.

**Scheme 1:**
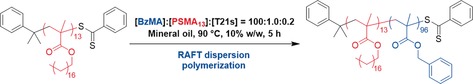
Synthesis of poly(stearyl methacrylate)_13_‐poly(benzyl methacrylate)_96_ (PSMA_13_‐PBzMA_96_) vesicles via RAFT dispersion polymerization of benzyl methacrylate at 10 % w/w solids in mineral oil at 90 °C.

Fielding et al. reported that poly(lauryl methacrylate)‐poly(benzyl methacrylate) (PLMA‐PBzMA) worms prepared in *n*‐dodecane undergo a worm‐to‐sphere transition upon heating as a result of surface plasticization of the PBzMA core‐forming block.14b,14c Similarly, the PSMA_13_‐PBzMA_96_ vesicles described in this study exhibited a vesicle‐to‐worm morphological transition on heating up to 150 °C. In order to observe this change in nanoparticle morphology by TEM, a 10 % w/w dispersion of PSMA_13_‐PBzMA_96_ vesicles was equilibrated at 150 °C for 10 min and diluted with *n*‐dodecane at the same temperature to produce a 0.1 % w/w dispersion. This hot dilution protocol ensured that the worms were retained as a kinetically‐trapped morphology on cooling to 20 °C (see Figure 1 b). The temperature‐dependence of the vesicle‐to‐worm transition was then studied in detail using SAXS (Figure 2).

Representative SAXS patterns for 5.0 % w/w PSMA_13_‐PBzMA_96_ nanoparticles in mineral oil (Figure 2) recorded at 20 °C (red data), 90 °C (blue data) and 130 °C (green data) are very similar, indicating that the vesicular morphology is retained throughout this temperature range. Notably, the local minimum at high *q* (≈0.5 nm^−1^) gradually shifts to higher *q*, suggesting that the vesicle membrane thickness decreases from 8.8 nm to 7.5 nm over this temperature range. These observations are consistent with greater solvation of the BzMA residues near the block junction on heating, which reduces the effective volume fraction of this membrane‐forming block (hence reducing the mean membrane thickness). On heating to 135 °C, a shallower gradient is observed at low *q* (orange data) and the local minimum at high *q* is shifted to lower *q*. This indicates the onset of the vesicle‐to‐worm transition. SAXS patterns continued to evolve on further heating, with a pure worm phase eventually being obtained at 145 °C (pink data) and 150 °C (black data). This morphological assignment is based on a gradient of approximately −1 at low *q*, as well as the loss of the feature at *q*≈0.05 nm^−1^ which represents the overall vesicle diameter.

Fitting SAXS patterns acquired at 20 °C and 150 °C using well‐known vesicle^15^ and worm‐like micelle^16^ models enabled the mean overall vesicle diameter, *D*_out_, vesicle membrane thickness, *T*_m_, worm thickness, *T*_w_, and worm contour length, *L*_w_, to be determined (see Figure 3). At 150 °C, the local minimum at *q*≈0.5 nm^−1^ indicates that *T*_w_=14.5 nm, which is somewhat larger than the *T*_m_ observed at 20 °C (8.8 nm). Similar differences were also observed by Rank et al.,^17^ who reported that poly(2‐vinyl pyridine)_66_‐poly(ethylene oxide)_46_ (P2VP_66_‐PEO_46_) diblock copolymer vesicles formed worms on cooling from 25 °C to 4 °C. These P2VP_66_‐PEO_46_ vesicles exhibited a *T*_m_ of 12 nm at 25 °C compared to a *T*_w_ of 16 nm for worms formed from the same diblock copolymer at 4 °C. This was attributed to the interdigitated core‐forming blocks producing a more densely packed core within the vesicle membrane—with little or no interdigitation occurring for the corresponding worms.^17^ Geometric calculations based on the PSMA_13_‐PBzMA_96_ vesicle/worm dimensions determined from SAXS analysis indicate that, on average, each vesicle dissociates to form three worms on heating from 20 °C to 150 °C (see Supporting Information). This observation is in rather good agreement with the ratio of the mean aggregation numbers determined for the vesicles (9784) and worms (3130) calculated from the respective SAXS models (see Supporting Information).

**Figure 3:**
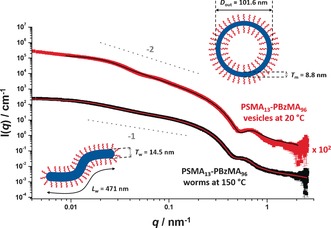
Representative SAXS patterns and fittings for PSMA_13_‐PBzMA_96_ vesicles at 20 °C (top, red data set plus black data fit) and PSMA_13_‐PBzMA_96_ worms at 150 °C (bottom, black data set plus red data fit). Inset cartoons indicate the mean overall vesicle diameter (*D*_out_), vesicle membrane thickness (*T*_m_), worm contour length (*L*_w_) and worm thickness (*T*_w_). Gradients of −2 and −1 are also shown as a guide to the eye.

The apparent degree of solvation of the PBzMA core‐forming block within the PSMA_13_‐PBzMA_96_ nano‐objects was monitored by variable temperature ^1^H NMR spectroscopy. First, the initial PSMA_13_‐PBzMA_96_ vesicles were transferred from mineral oil into *d*_26_‐dodecane via three centrifugation‐redispersion cycles (see Supporting Information). TEM studies of the final diluted copolymer dispersion in *n*‐dodecane confirmed that the vesicles survived this solvent exchange (see Figure S2). ^1^H NMR studies of 5.0 % w/w PSMA_13_‐PBzMA_96_ nano‐objects in *d*_26_‐dodecane were conducted from 25 to 150 °C (see Figure 4). This aliphatic solvent was selected because it is very similar to the mean chemical composition of mineral oil, which is not commercially available in deuterated form. The PBzMA benzylic proton signal “b” at 4.9 ppm became progressively more intense relative to the oxymethylene proton signal “a” of the PSMA block at 4.0 ppm, which confirms greater solvation of the PBzMA block. This indicates increasing solvent ingress into the PBzMA membranes at elevated temperatures, causing a change in the preferred diblock copolymer morphology via surface plasticization.14b Allowing for the known subtle differences in solvation between mineral oil and *n*‐dodecane,14c these NMR data are both physically reasonable and also consistent with the SAXS observations shown in Figure 2.

**Figure 4:**
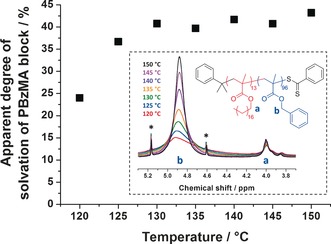
Temperature dependence of the apparent degree of solvation of PBzMA block as judged by variable temperature ^1^H NMR studies of a 5.0 % w/w PSMA_13_‐PBzMA_96_ nanoparticles in *d*_26_‐dodecane (see inset for assigned partial spectra from which these degrees of solvation were determined). Asterisks indicate satellite signals.

It is well‐known that dispersions of diblock copolymer worms form free‐standing gels at sufficiently high copolymer concentrations.^5^, ^18^ Moreover, a thermally‐triggered worm‐to‐sphere transition results in rapid in situ degelation.^13^, ^14^ A 10 % w/w dispersion of PSMA_13_‐PBzMA_96_ vesicles in mineral oil was studied to assess the effect of the vesicle‐to‐worm transition on its rheological behavior (see Figure 5). On heating from 20 °C to 130 °C, the storage (*G*′) and loss (*G*′′) moduli are reduced and *G*′ always remains lower than *G*′′, which indicates that the dispersion becomes less viscous. Such an inverse relationship between solution viscosity and temperature is well‐known for most fluids.^19^ However, on further heating from 130 to 135 °C, *G*′ increases by more than five orders of magnitude up to around 1 Pa. Thus the latter temperature corresponds to the critical gelation temperature (CGT), above which the dispersion behaves as a viscoelastic gel (since *G*′ now exceeds *G*′′). These observations are supported by the SAXS data shown in Figure 2, where PSMA_13_‐PBzMA_96_ vesicles are observed at temperatures between 20 °C and 130 °C, and the onset of the vesicle‐to‐worm transition occurred at around 135 °C. The first appearance of anisotropic worm‐like particles as judged by SAXS is in close agreement with the CGT determined by rheology, which strongly suggests that multiple inter‐worm contacts are responsible for the observed gelation. Moreover, varying the target mean degree of polymerization of the membrane‐forming PBzMA block from 85 to 100 enables the thermoresponsive behavior of the precursor vesicles to be tuned: an upturn in complex viscosity is observed over a 20 °C range (see Figure S3).

**Figure 5:**
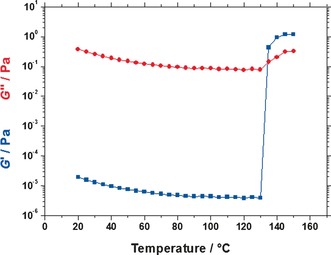
Dependence of the storage modulus (*G*′, blue data) and loss modulus (*G*′′, red data) of a 10 % w/w dispersion of PSMA_13_‐PBzMA_96_ nanoparticles in mineral oil upon heating from 20 to 150 °C. Data were obtained at 1.0 % strain using an angular frequency of 10 rad s^−1^, with a heating rate of 2 °C min^−1^.

There are rather few literature examples of significantly higher viscosities being achieved on increasing the solution temperature, with most of these studies involving aqueous formulations.^13^, ^20^ However, these thermal transitions typically occur at relatively low temperatures (4 °C to 30 °C) and usually increase the aqueous solution viscosity by less than an order of magnitude. As far as we are aware, there is just one literature example of an aqueous formulation that is directly analogous to the thermal transition described herein: certain ionic surfactant vesicles can form a highly viscous dispersion of anisotropic micelles on heating to 60 °C.^21^ Of perhaps more relevance to the present study, oil thickening has been widely reported for low molecular weight gelators.^22^ However, such gelators are usually heated to obtain a homogeneous solution, with gelation then occurring on cooling. Thus our observations suggest that diblock copolymer vesicles can provide a new high‐temperature oil‐thickening mechanism to rival that recently achieved using thermosensitive graft copolymers.^23^

In summary, PSMA_13_‐PBzMA_96_ vesicles prepared directly in mineral oil via PISA undergo an order‐order morphological transition on heating from 20 °C to 150 °C. This system has been characterized using TEM, SAXS, rheology and variable temperature ^1^H NMR spectroscopy. The latter technique confirms that greater solvation of the PBzMA core‐forming block occurs at elevated temperatures. Such surface plasticization triggers the observed morphological transition above 135 °C, as indicated by SAXS, TEM and rheology studies. This vesicle‐to‐worm transition provides a unique high‐temperature oil‐thickening mechanism that may offer some commercial utility.

## Conflict of interest

The authors declare no conflict of interest.

## Supporting information

As a service to our authors and readers, this journal provides supporting information supplied by the authors. Such materials are peer reviewed and may be re‐organized for online delivery, but are not copy‐edited or typeset. Technical support issues arising from supporting information (other than missing files) should be addressed to the authors.

Supplementary
