# Supplementary material for: A Vesicle‐to‐Worm Transition Provides a New High‐Temperature Oil Thickening Mechanism
Source: Angew Chem Int Ed Engl. 2017 Jan 10;56(7):1746–50. doi: 10.1002/anie.201609365 (PMC5396375; doi:10.1002/anie.201609365)
Supplement: Supplementary file 1 — Supplementary [file ANIE-56-1746-s001.pdf]

Angewandte Chemie
Eine Zeitschrift der Gesellschaft Deutscher Chemiker

Supporting Information

A Vesicle-to-Worm Transition Provides a New High-Temperature Oil Thickening Mechanism

Matthew J. Derry, Oleksandr O. Mykhaylyk, and Steven P. Armes**

anie_201609365_sm_miscellaneous_information.pdf

Experimental Section

Materials

Monomers were purchased from Sigma-Aldrich (UK) and passed through basic alumina prior to use. $CDCl_3$ and all other reagents were purchased from Sigma-Aldrich (UK) and were used as received, unless otherwise noted. THF and toluene were purchased from Fisher Scientific (UK), CD_2Cl_2 and d_{26}-dodecane were purchased from Goss Scientific (UK) and industrial mineral oil was provided by Lubrizol Ltd.

Synthesis of poly(stearyl methacrylate) macro-chain transfer agent (PSMA macro-CTA)

The $PSMA_{13}$ macro-CTA used in the present work was synthesized as follows. Stearyl methacrylate (SMA; 30.6 g; 90.5 mmol), cumyl dithiobenzoate (CDB; 4.93 g, 18.1 mmol), 2,2'-azobisisobutyronitrile (AIBN; 594 mg, 3.6 mmol; CDB/AIBN molar ratio = 5.0) and toluene (54.2 g). The sealed reaction vessel was purged with nitrogen and placed in a pre-heated oil bath at 70 °C for 10 h. The resulting PSMA (SMA conversion = 76 %; M_n = 4900 g mol^{-1}, M_w = 5700 g mol^{-1}, M_w/M_n = 1.17) was purified by precipitation into excess ethanol. The mean degree of polymerization (DP) of this macro-CTA was calculated to be 13 using ^1H NMR spectroscopy by comparing the integrated signals corresponding to the CDB aromatic protons at 7.0-7.5 ppm with that assigned to the two oxymethylene protons of PSMA at 3.4-4.2 ppm.

Synthesis of poly(stearyl methacrylate)-poly(benzyl methacrylate) (PSMA-PBzMA) diblock copolymer vesicles

A typical RAFT non-polar dispersion polymerization synthesis of $PSMA_{13}$-$PBzMA_{96}$ diblock copolymer vesicles at 10% w/w solids was carried out as follows. Benzyl methacrylate (BzMA; 1.89 g; 10.7 mmol), *tert*-butyl peroxy-2-ethylhexanoate initiator (T21s; 4.62 mg; 21.4 μmol; dissolved at 10.0% v/v in mineral oil) and $PSMA_{13}$ macro-CTA (0.5 g; 107 μmol; macro-CTA/initiator molar ratio = 5.0) were dissolved in mineral oil (21.5 g). The reaction mixture was sealed in a 100 mL round-bottomed flask and purged with nitrogen gas for 30 min. The deoxygenated solution was then placed in a pre-heated oil bath at 90 °C for 4 h (final BzMA conversion = 97 %; M_n = 16 600 g mol^{-1}, M_w/M_n = 1.16).

Gel permeation chromatography

Molecular weight distributions were assessed by gel permeation chromatography (GPC) using THF eluent. The THF GPC system was equipped with two 5 μm (30 cm) Mixed C columns; a WellChrom K-2301 refractive index detector operating at 950 ± 30 nm. The mobile phase contained 2.0% v/v triethylamine and 0.05% w/v butylhydroxytoluene (BHT) with a toluene flow rate marker and the flow rate was fixed at 1.0 mL min^{-1}. A series of ten near-monodisperse poly(methyl methacrylate) standards (M_p values ranging from 1280 to 330 000 g mol^{-1}) were used for calibration.

^1H NMR spectroscopy

^1H NMR spectra were recorded in either CD_2Cl_2 or $CDCl_3$ using a Bruker AV1-400 or AV1-250 MHz spectrometer. Typically 64 scans were averaged per spectrum. For variable temperature ^1H NMR studies, the 10% w/w dispersion of $PSMA_{13}$-$PBzMA_{96}$ vesicles was centrifuged for at 13 000 rpm for 1 h. The sedimented vesicles were then redispersed and the centrifugation step repeated until three cycles were completed. Finally, the sedimented vesicles were redispered using d_{26}-dodecane to produce a 5.0% w/w dispersion. Variable temperature ^1H NMR spectra were then recorded from 120 to 150 °C (32 scans per spectrum) using a Bruker Avance III HD spectrometer operating at 500.13 MHz. Spectra were collected over a spectral window of 25 kHz, with an acquisition time of 4 s and allowing for a relaxation delay time of 15 s between each scan. For data analysis, a 2 Hz line broadening was applied to all spectra before performing peak deconvolution between 5.5 and 3.5 ppm using Bruker TopSpin NMR software. Peak areas were used to determine the apparent solvation of the core-forming PBzMA block by comparing the oxymethylene signal assigned to the PSMA stabiliser with the benzylic protons of the PBzMA core-forming block (see Figure 4 inset).

Transmission electron microscopy

Transmission electron microscopy (TEM) studies were conducted using a Philips CM 100 instrument operating at 100 kV and equipped with a Gatan 1 k CCD camera. Diluted block copolymer solutions (0.10% w/w) were placed on carbon-coated copper grids and exposed to ruthenium(VIII) oxide vapor for 7 min at 20 °C prior to analysis.[1] This heavy metal compound acted as a positive stain for the core-forming PBzMA block to improve contrast. The ruthenium(VIII) oxide was prepared as follows: ruthenium(IV) oxide (0.30 g) was added to water (50 g) to form a black slurry; addition of sodium periodate (2.0 g) with stirring produced a yellow solution of ruthenium(VIII) oxide within 1 min.

Rheology measurements

An Anton Paar MCR 502 rheometer equipped with a variable temperature Peltier plate and hood, and a 50 mm 2° stainless steel cone was used for all experiments. The loss and storage moduli were measured as a function of temperature at a fixed strain of 1.0% and a shear rate of 10 rad s^{-1} and a heating rate of 2 °C min^{-1}.

Small-angle X-ray scattering

SAXS patterns were collected at a synchrotron source (ESRF, station ID02, Grenoble, France) using monochromatic X-ray radiation (wavelength λ = 0.0995 nm, with q ranging from 0.003 to 2.5 nm^{-1}, where $q = 4\pi \sin \theta/\lambda$ is the length of the scattering vector and θ is one-half of the scattering angle) and a Ravonix MX-170HS CCD detector. Glass capillaries of 2 mm diameter were used as a sample holder and the sample temperature was controlled using a HFSX350-CAP heating/cooling capillary holding stage (Linkam Scientific Instruments Ltd., Tadworth, UK), with 2 min equilibration before data collection. Scattering data were reduced using standard routines from the beamline and were further analyzed using Irena SAS macros for Igor Pro.[2] Water was used for the absolute intensity calibration. Measurements were conducted on a 5.0% w/w dispersion of $PSMA_{13}$-$PBzMA_{96}$ particles in mineral oil.

Geometric calculations for the vesicle-to-worm transition

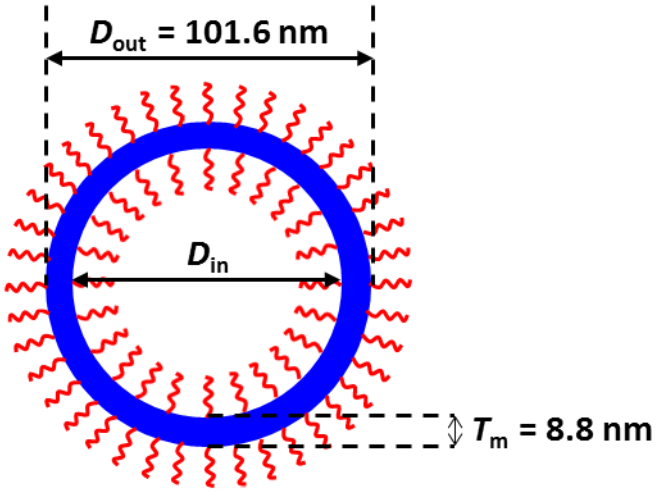

$$D_{\mathrm{in}} = D_{\mathrm{out}} - 2T_{\mathrm{m}}$$

$$D_{\mathrm{in}} = 101.6\ \mathrm{nm} - (2 \times 8.8\ \mathrm{nm}) = 84.0\ \mathrm{nm}$$

$$\textit{Volume of the vesicle membrane}, V_{\mathrm{vm}} = V_{\mathrm{out}} - V_{\mathrm{in}} = \frac{4}{3}\pi\left[\left(\frac{D_{\mathrm{out}}}{2}\right)^3 - \left(\frac{D_{\mathrm{in}}}{2}\right)^3\right]$$

$$V_{\mathrm{vm}} = \frac{4}{3}\pi\left[\left(\frac{101.6\ \mathrm{nm}}{2}\right)^3 - \left(\frac{84.0\ \mathrm{nm}}{2}\right)^3\right] = 2.39 \times 10^5\ \mathrm{nm}^3$$

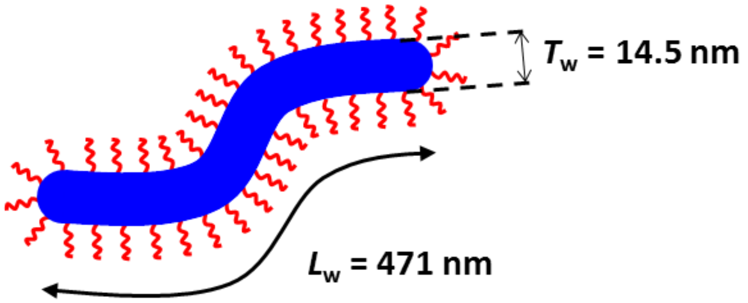

$$Volume\ of\ the\ worm\ core, V_{wc} = \left[\pi\left(\frac{T_w}{2}\right)^2 L_w\right] + \left[\frac{4}{3}\pi\left(\frac{T_w}{2}\right)^3\right]$$

$$V_{wc} = \left[\pi\left(\frac{14.5\text{ nm}}{2}\right)^2 \times 471\text{ nm}\right] + \left[\frac{4}{3}\pi\left(\frac{14.5\text{ nm}}{2}\right)^3\right] = 7.94 \times 10^4\text{ nm}^3$$

$$Average\ number\ of\ worms\ formed\ from\ one\ vesicle = \frac{V_m}{V_w} = \frac{2.39 \times 10^5\text{ nm}^3}{7.94 \times 10^4\text{ nm}^3} = 3.01 \sim 3$$

Comparison of aggregation numbers for the vesicle-to-worm transition

$$Mean\ vesicle\ aggregation\ number, N_v = \frac{V_{vm}}{V_m} = \frac{2.39{\times}10^5\text{nm}^3}{24.426\text{ nm}^3} = 9784$$

$$Mean\ worm\ aggregation\ number, N_w = \frac{V_{wc}}{V_s} = \frac{7.94{\times}10^4\text{nm}^3}{24.426\text{ nm}^3} = 3130$$

$$Average\ number\ of\ worms\ formed\ from\ one\ vesicle = \frac{N_v}{N_w} = \frac{9{,}784}{3{,}130} = 3.1$$

Figures

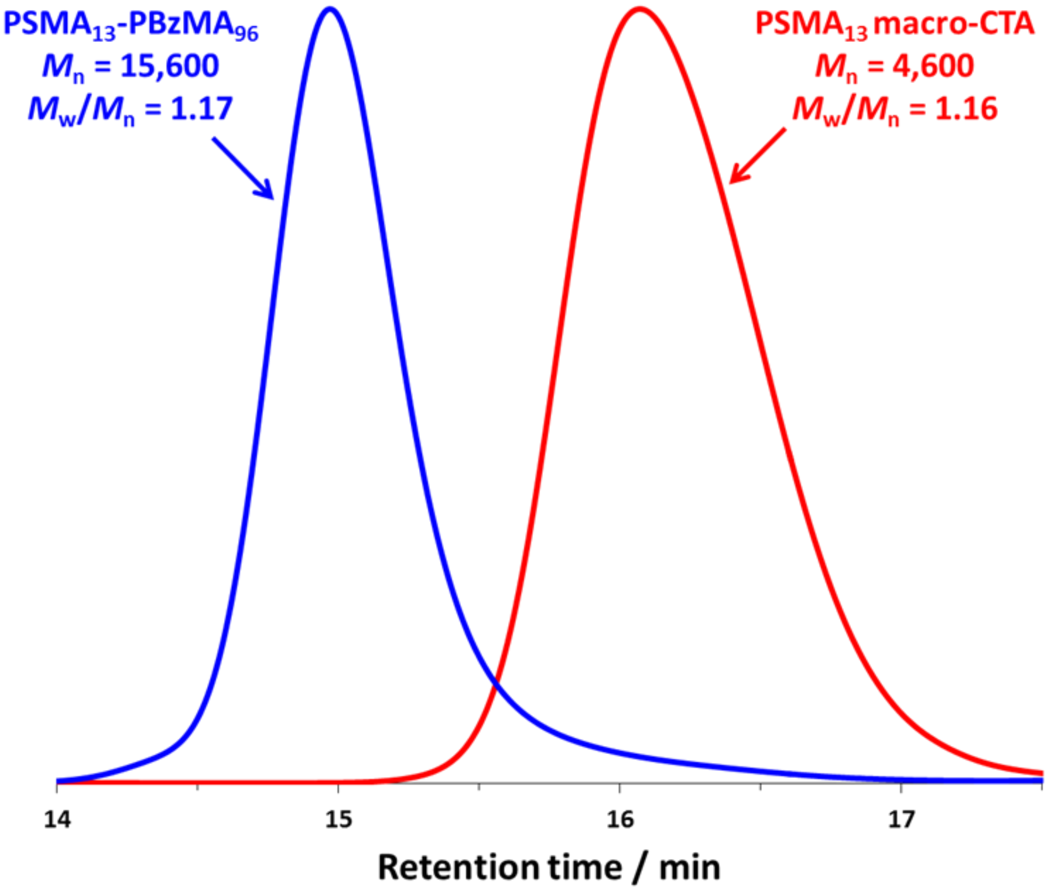

Figure S1. Gel permeation chromatograms (vs. poly(methyl methacrylate) standards) obtained for the vesicle-forming $PSMA_{13}$-$PBzMA_{96}$ diblock copolymers synthesized via RAFT dispersion polymerization in mineral oil at 90 °C and 10% w/w solids. The $PSMA_{13}$ macro-CTA (prepared in toluene at 70 °C at 40% w/w solids) is also shown as a reference.

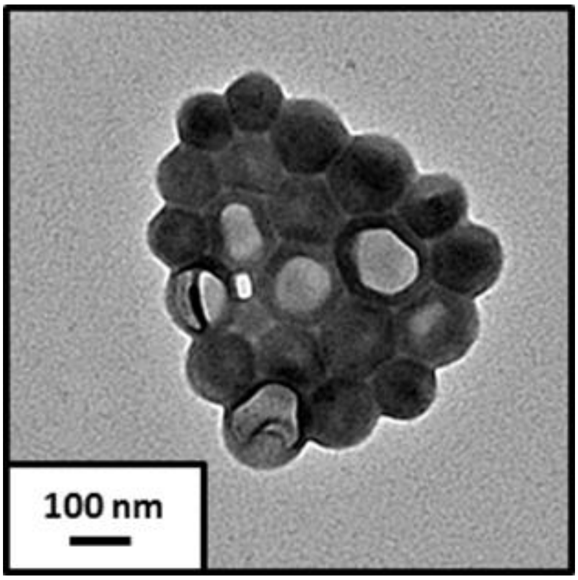

Figure S2. Transmission electron micrograph obtained for $PSMA_{13}$-$PBzMA_{96}$ vesicles in *n*-dodecane after three centrifugation-redispersion cycles (centrifugation at 13,000 rpm for 60 min).

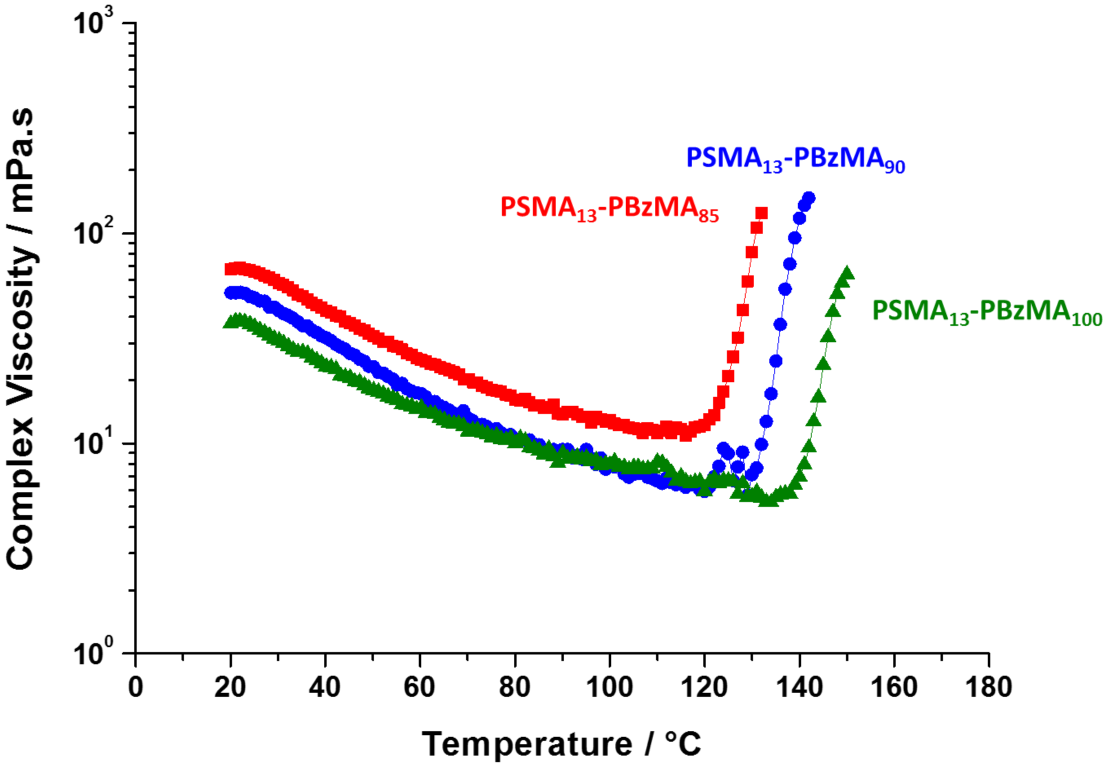

Figure S3. Complex viscosity vs. temperature plots for $PSMA_{13}$-$PBzMA_{85}$ (red squares), $PSMA_{13}$-$PBzMA_{90}$ (blue circles) and $PSMA_{13}$-$PBzMA_{100}$ (green triangles) nanoparticles dispersed in mineral oil at 10% w/w solids. Data were obtained at 1.0% strain using an angular frequency of 10 rad s^{-1} at a heating rate of 2 °C min^{-1}.

SAXS models

In general, the X-ray intensity scattered by a dispersion of nano-objects, I(q), can be expressed as:

$$I(q) = N.S(q) \int_0^{\infty} \dots \int_0^{\infty} F(q, r_1, \dots, r_k)^2 \Psi(r_1, \dots, r_k) dr_1 \dots dr_k \qquad (S1)$$

where $F(q, r_1, \dots, r_k)$ is their form factor, $r_1, \dots, r_k$ is a set of k parameters describing the structural morphology, $\Psi(r_1, \dots, r_k)$ is the distribution function, S(q) is the structure factor and N is the nano-object number density per unit volume expressed as:

$$N = \frac{\varphi}{\int_0^{\infty} \dots \int_0^{\infty} V(r_1, \dots, r_k) \Psi(r_1, \dots, r_k) dr_1 \dots dr_k} \qquad (S2)$$

where $V(r_1, \dots, r_k)$ is volume of the nano-object and φ is their volume fraction in the dispersion.

Vesicle model

The vesicle form factor in Equation S1 is expressed as:[3]

$$F_{\text{ves}}(q) = N_{\text{v}}^2 \beta_{\text{m}}^2 A_{\text{m}}^2(q) + N_{\text{v}} \beta_{\text{vc}}^2 F_{\text{c}}(q, R_{\text{g}}) + N_{\text{v}}(N_{\text{v}} - 1) \beta_{\text{vc}}^2 A_{\text{vc}}^2(q) + 2 N_{\text{v}}^2 \beta_{\text{m}} \beta_{\text{vc}} A_{\text{m}}(q) A_{\text{vc}}(q) \qquad (S3)$$

The X-ray scattering length contrast for the membrane-forming block (PBzMA) and the coronal stabiliser block (PSMA) is given by $\beta_{\text{m}} = V_{\text{m}}(\xi_{\text{m}} - \xi_{\text{sol}})$ and $\beta_{\text{vc}} = V_{\text{vc}}(\xi_{\text{vc}} - \xi_{\text{sol}})$, respectively, where ξ_{m}, ξ_{vc} and ξ_{sol} are the X-ray scattering length densities of the membrane-forming block (ξ_{PBzMA} = 10.38 x 10^{10} cm^{-2}), the coronal stabiliser block (ξ_{PSMA} = 9.24 x 10^{10} cm^{-2}) and the solvent (ξ_{sol} = 7.63 x 10^{10} cm^{-2}). V_{m} and V_{vc} are the volumes of the membrane-forming block and the coronal stabiliser block, respectively. The volumes were obtained from $V = \frac{M_{\text{n,pol}}}{N_{\text{A}}\rho}$ using the density of PBzMA (ρ_{PBzMA} = 1.15 g cm^{-3})[4] and the solid-state homopolymer density of PSMA determined by helium pycnometry (ρ_{PSMA} = 0.97 g cm^{-3}), where $M_{\text{n,pol}}$ corresponds to the number-average molecular weight of the block determined by ^{1}H NMR spectroscopy. The amplitude of the membrane self-term is:

$$A_{\text{m}}(q) = \frac{V_{\text{out}} \varphi(qR_{\text{out}}) - V_{\text{in}} \varphi(qR_{\text{in}})}{V_{\text{out}} - V_{\text{in}}} \exp\left(-\frac{q^2 \sigma_{\text{in}}^2}{2}\right) \qquad (S4)$$

where $R_{\text{in}} = R_{\text{m}} - \frac{1}{2}T_{\text{m}}$ is the inner radius of the membrane, $R_{\text{out}} = R_{\text{m}} + \frac{1}{2}T_{\text{m}}$ is the outer radius of the membrane, $V_{\text{in}} = \frac{4}{3}\pi R_{\text{in}}^3$, $V_{\text{out}} = \frac{4}{3}\pi R_{\text{out}}^3$. It should be noted that Equation S3 differs from the original work in which they were first described.[3] The exponent term in Equation S4 represents a sigmoidal interface between the blocks, with a width σ_{in} accounting for a decaying scattering length density at the membrane surface. The value of σ_{in} was fixed at 2.5. The mean vesicle aggregation number, N_{v}, is given by:

$$N_{\text{v}} = (1 - x_{\text{sol}}) \frac{V_{\text{out}} - V_{\text{in}}}{V_{\text{m}}} \qquad (S5)$$

where x_{sol} is the solvent (i.e. mineral oil) volume fraction within the vesicle membrane. Assuming that there is no penetration of the solvophilic coronal blocks into the solvophobic membrane, the amplitude of the vesicle corona self-term is expressed as:

$$A_{\text{vc}}(q) = \Psi(qR_{\text{g}})\frac{1}{2}\left[\frac{\sin[q(R_{\text{out}}+R_{\text{g}})]}{q(R_{\text{out}}+R_{\text{g}})} + \frac{\sin[q(R_{\text{in}}-R_{\text{g}})]}{q(R_{\text{in}}-R_{\text{g}})}\right] \quad (S6)$$

where the term outside the square brackets is the factor amplitude of the corona block polymer chain such that:

$$\Psi(qR_{\text{g}}) = \frac{1-\exp(-qR_{\text{g}})}{(qR_{\text{g}})^2} \quad (S7)$$

The obtained R_g of the $PSMA_{13}$ coronal block of ~1.21 nm is comparable to the estimated value of 0.92 nm. The latter can be calculated from the total contour length of the $PSMA_{13}$ block, L_{PSMA13} = 13 x 0.255 nm = 3.315 nm (since the projected contour length per SMA monomer repeat unit is defined by two carbon bonds in an all-*trans* conformation, or 0.255 nm) and the Kuhn length of 1.53 nm [based on the known literature value for PMMA[5]] result in an approximate R_g of $(3.315 \text{ x } 1.53/6)^{1/2}$ = 0.92 nm. It was assumed for the vesicle model that two parameters are polydisperse: the overall radius of the vesicles and the membrane thickness (R_{m} and T_{m}, respectively). They are considered to have a Gaussian distribution and, therefore, the polydispersity function in Equation S1 can be expressed as:

$$\Psi(r_1, r_2) = \frac{1}{\sqrt{2\pi\sigma_{R\text{m}}^2}}\exp\left(-\frac{(r_1 - R_{\text{m}})^2}{2\sigma_{R\text{m}}^2}\right)\frac{1}{\sqrt{2\pi\sigma_{T\text{m}}^2}}\exp\left(-\frac{(r_1 - T_{\text{m}})^2}{2\sigma_{T\text{m}}^2}\right) \quad (S8)$$

where $\sigma_{R\text{m}}$ and $\sigma_{T\text{m}}$ are the standard deviations for R_{m} and T_{m}, respectively. Following Equation S2 the number density per unit volume for the vesicle model is expressed as:

$$N = \frac{\varphi}{\int_0^\infty \int_0^\infty V(r_1, r_2)\Psi(r_1, r_2)dr_1 dr_2} \quad (S9)$$

where φ is the total *volume fraction* of copolymer in the vesicles and $V(r_1, r_2)$ is the total *volume* of copolymers in a vesicle $[V(r_1, r_2) = (V_{\text{m}} + V_{\text{vc}})N_{\text{v}}(r_1, r_2)]$.

Worm-like micelle model

The worm-like micelle form factor for Equation S1 is given by:[6]

$$F_{\text{w\_mic}}(q) = N_{\text{w}}^2\beta_{\text{s}}^2F_{\text{sw}}(q) + N_{\text{w}}\beta_{\text{c}}^2F_{\text{c}}(q, R_{\text{g}}) + N_{\text{w}}(N_{\text{w}}-1)\beta_{\text{c}}^2S_{\text{cc}}(q) + 2N_{\text{w}}^2\beta_{\text{s}}\beta_{\text{c}}S_{\text{sc}}(q) \quad (S10)$$

where the core block and the corona block X-ray scattering length contrast is given by $\beta_{\text{s}} = V_{\text{s}}(\xi_{\text{s}} - \xi_{\text{sol}})$ and $\beta_{\text{c}} = V_{\text{c}}(\xi_{\text{c}} - \xi_{\text{sol}})$, respectively. Here ξ_{s}, ξ_{c} and ξ_{sol} are the X-ray scattering length densities of the core block (ξ_{PBzMA} = 10.38 x 10^{10} cm^{-2}), the corona block (ξ_{PSMA} = 9.24 x 10^{10} cm^{-2}) and the solvent (ξ_{sol} = 7.63 x 10^{10} cm^{-2}), respectively. V_{s} and V_{c} are volumes of the core block (V_{PBzMA}) and the corona block (V_{PSMA}), respectively. Using the molecular weights of the PBzMA and PSMA blocks and their respective mass densities: ρ_{PBzMA} = 1.15 g cm^{-3} and ρ_{PSMA} = 0.97 g cm^{-3}, the individual block volumes can be calculated from $V = \frac{M_{\text{n,pol}}}{N_{\text{A}}\rho}$, where $M_{\text{n,pol}}$ corresponds to the number-average molecular weight of the block determined by ^{1}H NMR spectroscopy. The self-correlation term for the worm-like micelle core or radius R_{sw} is:

$$F_{\text{sw}}(q) = F_{\text{worm}}(q, L_{\text{w}}, b_{\text{w}})A_{\text{cs}_{\text{worm}}}{}^2(q, R_{\text{sw}}) \quad (S11)$$

which is a product of a core cross-section term:

$$F_{\mathrm{cs}_{\mathrm{worm}}}(q, R_{\mathrm{g}}) = A_{\mathrm{cs}_{\mathrm{worm}}}{}^{2}(q, R_{\mathrm{sw}}) = \left[2\frac{J_1(qR_{\mathrm{sw}})}{qR_{\mathrm{sw}}}\right]^2 \qquad (S12)$$

where J_1 is the first-order Bessel function of the first kind, and a form factor $F_{\mathrm{worm}}(q, L_{\mathrm{w}}, b_{\mathrm{w}})$ for self-avoiding semi-flexible chains represents the worm-like micelle, where b_{w} is the worm Kuhn length and L_{w} is the mean worm contour length. A complete expression for the chain form factor can be found elsewhere.[7] The self- correlation term for the corona block is given by the Debye function:

$$F_{\mathrm{c}}(q, R_{\mathrm{g}}) = \frac{2\left[\exp\left(-q^2 R_{\mathrm{g}}{}^2\right) - 1 + q^2 R_{\mathrm{g}}{}^2\right]}{q^4 R_{\mathrm{g}}{}^4} \qquad (S13)$$

where R_{g} is the radius of gyration of the PSMA coronal block. The mean aggregation number of the worm-like micelle is given by:

$$N_{\mathrm{w}} = (1 - x_{\mathrm{sol}})\frac{\pi R_{\mathrm{sw}}{}^2 L_{\mathrm{w}}}{V_{\mathrm{s}}} \qquad (S14)$$

where x_{sol} is the volume fraction of solvent within the worm-like micelle core. Possible semi-spherical caps at the ends of each worm are not considered in this form factor.

References

[1] J. S. Trent, *Macromolecules*, **1984**, *17*, 2930-2931.

[2] J. Ilavsky, P. R. Jemain, *Journal of Applied Crystallography*, **2009**, *42*, 347-353.

[3] J. Bang, S. M. Jain, Z. B. Li, T. P. Lodge, J. S. Pedersen, E. Kesselman, Y. Talmon, *Macromolecules* **2006**, *39*, 1199-1208.

[4] L. A. Fielding, J. A. Lane, M. J. Derry, O. O. Mykhaylyk, S. P. Armes, *Journal of the American Chemical Society*, **2014**, *136*, 5790-5798.

[5] L. J. Fetters, D. J. Lohsey, R. H. Colby, *in Physical Properties of Polymers Handbook, ed J. E. Mark, Springer, New York, 2nd Ed,* **2007**, *25*, 447.

[6] J. S. Pedersen, *Journal of Applied Crystallography*, **2000**, *33*, 637-640.

[7] J. S. Pedersen, P. Shurtenberger, *Macromolecules*, **1996**, *29*, 7602-7612.
